# Assessment of Thymic Output Dynamics After *in utero* Infection of Mice With Coxsackievirus B4

**DOI:** 10.3389/fimmu.2020.00481

**Published:** 2020-04-02

**Authors:** Aymen Halouani, Habib Jmii, Gwennaëlle Bodart, Hélène Michaux, Chantal Renard, Henri Martens, Mahjoub Aouni, Didier Hober, Vincent Geenen, Hela Jaïdane

**Affiliations:** ^1^Laboratoire des Maladies Transmissibles et Substances Biologiquement Actives, Faculté de Pharmacie de Monastir, Université de Monastir, Monastir, Tunisia; ^2^Faculté des Sciences de Tunis, Université de Tunis El Manar, Tunis, Tunisia; ^3^GIGA-I^*3*^ Neuroimmunoendocrinology, GIGA Research Institute, University of Liège, Liège, Belgium; ^4^Université de Lille, CHU de Lille, Laboratoire de Virologie, Lille, France

**Keywords:** thymus, T cell, thymic export, T cell receptor (TCR) rearrangement excision circles, autoimmune diseases, *Ptk7* gene, coxsackievirus B, vertical transmission

## Abstract

The thymus is the main organ of the lymphatic system, in which T cells undergo a rigorous selection to ensure that their receptors (TCRs) will be functional and will not react against the self. Genes encoding for TCR chains are fragmented and must be rearranged by a process of somatic recombination generating TCR rearrangement excision circles (TRECs). We recently documented coxsackievirus B4 (CV-B4) infection of Swiss albino mouse thymus in the course of *in utero* transmission. In the current study, we intended to evaluate thymic output in this experimental model. For this purpose, pregnant Swiss albino mice were inoculated with CV-B4 at day 10 or 17 of gestation, and thymus and spleen were sampled from offspring at different time points and then subjected to quantification of TREC molecules and *Ptk7* gene expression. Results showed a pronounced effect of *in utero* CV-B4 infection on the thymus with an increase in the cellularity and, consequently, the weight of the organ. sj and DβTREC analysis, by real-time PCR, revealed a significant decrease following CV-B4 infection compared to controls, a decrease which gets worse as time goes by, both in the thymus and in the periphery. Those observations reflect a disturbance in the export of T cells to the periphery and their accumulation within the thymus. The evaluation of *Ptk7* transcripts in the thymus, for its part, showed a decrease in expression, especially following an infection at day 10 of gestation, which supports the hypothesis of T cell accumulation in a mature stage in the thymus. The various effects observed correlate either negatively or positively with the viral load in the thymus and spleen. Disruption in thymic export may indeed interfere with T cell maturation. We speculate that this may lead to a premature release of T cells and the possibility of circulating autoreactive or proliferation-impaired T cell clones.

## Introduction

The thymus is an asymmetrical bilobate gland localized in the upper chest region above the pericardium of the heart and arises from the third pharyngeal pouch of the endoderm [reviewed by Blackburn and Manley (1)]. The thymus is a primary lymphoid organ, in which T lymphocytes or T cells undergo a rigorous selection to ensure that they will not react against the self and that their receptors are functional. This process of maturation and education of thymocytes (intrathymic T cells) consists of the expression of a functional T cell receptor (TCR) on lymphoid precursors from the bone marrow (BM) and fetal liver to result in the formation of mature T cells (so-called naive) that gain the periphery [reviewed by Zhang and Bhandoola (2)]. TCRα and β-chain gene are composed of Variable (V), Diversity (D, only in TCR β-chain gene), and Joining (J) gene segments. This process called V(D)J recombination takes place in the thymus during the maturation of lymphocyte precursors and involves enzymes expressed only in lymphocytes, the recombination-activating genes (RAG)-1 and RAG-2 (3). The β-chain gene rearrangement occurs first and begins at the double negative (DN) 2 stage. It requires the successful recombination of D and J gene segments, followed by V to DJ gene segment recombination (3). The rearrangement of the TCR α-chain locus occurs at the double positive (DP) stage (3).

Excisional rearrangements of TCR genes in thymocytes generate episomal DNA circles produced in thymocytes and called TCR rearrangement excision circles (TRECs). TRECs are stable, not replicated during mitosis, progressively diluted with cell division, and therefore enriched in thymocytes, recent thymic emigrants (RTEs), and naive T cells (4).

TRECs are formed during any TCR gene rearrangement, but classically only two types are quantified to assess thymic function: DJβTRECs (also called DβTRECs) formed during β chain rearrangement and sjTRECs created by the excision of the δ locus located inside the α-chain locus (5). As sjTRECs are produced at the DP stage, they are found in almost each cell leaving the thymus, known as RTEs, and are therefore a good marker of the quantity of cells exported by the thymus. Conversely, DβTRECs are generated just before the intensive proliferation phase (5). By calculating the ratio of sj/Dβ TRECs, the extent of this intrathymic proliferation can thus be assessed (6).

TRECs have been considered for a long time as the best tool to investigate thymic function (7). Recently, protein tyrosine kinase 7 (PTK7), also known as colon carcinoma kinase-4, has been identified as a novel surface marker for RTEs (8). Intrathymic PTK7 expression progressively decreases with maturation until it is lost on mature SP CD8 T cells before and on SP CD4 T cells shortly after exportation (8–10).

A reduced thymic activity was one of the common clinical features in patients suffering from different autoimmune diseases, namely, rheumatoid arthritis (RA) (11, 12) with a lower TREC number especially in isolated CD4^+^ T cells (13), multiple sclerosis with a decrease in TREC frequencies in both CD4^+^ and CD8^+^ T cells (14, 15), systemic lupus erythematosus (SLE) (15–17), and myasthenia gravis (18). These data suggest that a disruption in thymic export might interfere with T cell maturation and selection process, resulting in a premature output of autoreactive or proliferation-impaired T cell clones (11).

The thymus is mostly active during the fetal period and childhood and then begins to decrease in size and continues to shrink with age, together with replacement of the epithelial tissue by fat and connective tissue (19).

Group B coxsackieviruses (CV-B) belong to the *Enterovirus B* species, *Enterovirus* genus, *Picornaviridae* family. The virus particle is small, non-enveloped, and consists of a single-stranded RNA genome with positive polarity encompassed in an icosahedral capsid. The genome comprises a single open-reading frame flanked by two untranslated regions at both the 5′ and 3′ ends [reviewed by Tracy et al., (20)]. Since CV-B discovery, arguments of its association with induction or acceleration of autoimmunity are accumulating. CV-B infections may be involved in a variety of acute and chronic diseases that have an autoimmune component, including myocarditis, dilated cardiomyopathy, type 1 diabetes (T1D), encephalitis, thyroiditis, or Sjögren's syndrome [reviewed by Jaïdane et al., (21)].

In previous studies, CV-B4 infection of *in vitro*-cultured murine thymic cells (22), human (23) and murine thymic epithelial cells (24), human (25) and murine fetal thymic organ cultures (FTOCs) (26), and the thymus of Swiss albino mice inoculated through the oral route (27) was documented with various effects. Briefly, the diabetogenic CV-B4 E2 persists in primary cultures of human TECs with an increase of interleukin (IL)-6, leukemia inhibitory factor (LIF), and granulocyte-macrophage colony-stimulating factor (GM-CSF) (23). CV-B4 E2 is also able to infect human FTOC, inducing upregulation of major histocompatibility complex (MHC) class I molecules and severe depletion of thymocytes with anomalies in their subpopulations (25). CV-B4 infection of murine FTOC also disturbs the T lymphocyte maturation/differentiation process (26). Chatterjee et al. (28) previously noticed such abnormalities in thymocyte populations following inoculation of 5- to 6-week-old male SJL/J mice by the same strain. Interestingly, CV-B4 E2 is able to reach the thymus during a systemic infection of outbred Swiss albino mice inoculated through the oral route, and its genome remains detectable up to 70 days post-inoculation (27). CV-B4 E2, the prototype CV-B4 JVB, CV-B3, and echovirus 1 are able to persistently infect a murine TEC line with a drastic decrease in the expression of insulin-like growth factor-2 (24).

Since thymic activity begins during fetal life, it is very likely that a viral infection of the thymus could lead to its dysfunction. Indeed, we recently demonstrated that CV-B4 E2 infects the thymus in the course of *in utero* virus transmission, which induces significant anomalies in thymic T cell subsets (21). In this study, we used the same model of Swiss albino pregnant mice to scrutinize the impact of *in utero* CV-B4 E2 infection on thymic export by measuring different parameters.

## Materials and Methods

### Virus

CV-B4 E2 strain (kindly provided by J. W. Yoon, Julia McFarlane Diabetes Research Centre, Calgary, Alberta, Canada) was propagated in human epithelial type 2 (HEp-2) cells (BioWhittaker, Walkersville, MD, USA) in Eagle's minimum essential medium (MEM; GIBCO BRL, Invitrogen, Gaithersburg, MD, USA) supplemented with 10% heat-inactivated fetal calf serum (FCS; GIBCO BRL), 2 mM L-glutamine (BioWhittaker), 50 μg/ml streptomycin, 50 IU/ml penicillin (GIBCO BRL), and 2.5 μg/ml Fungizone (amphotericin B; Apothecon). Supernatants were collected 3 days after, submitted to three cycles of freezing/thawing, then clarified by centrifugation at 4,000 × g for 10 min, divided into aliquots and stored at −80°C. Viral titers in stocks were determined on HEp-2 cells by limiting dilution assay for 50% tissue culture infectious dose (TCID_50_) by the method of Reed and Muench (29).

### Mating Protocol and Mice Inoculation

Eight- to 10-week-old female and male Swiss albino mice were bred under standard conditions, with *ad libitum* water and food. Four females per male were caged together, and the presence of a vaginal plug was checked three times a day. The day the vaginal plug was observed was considered as the first day of gestation (day 1G).

Female mice were orally inoculated on the 10th or 17th day of pregnancy with 2 × 10^6^ TCID_50_ of CV-B4 E2 contained in 200 μl MEM. Age-matched offspring from naive pregnant mice served as mock-infected negative controls. Offspring were weighed and euthanized with isoflurane at different time points [near term (day 17G), the day of birth (referred to as day 1), and day 5 after birth]. Thymus and spleen were removed, washed with cold phosphate buffered saline (PBS), and weighed and treated for TRECs and *Ptk7* mRNA quantification. Each thymus was cut into two halves: the first half (pool of three halves of three different thymuses from three mice of the same litter) was subjected to thymocyte isolation and then DNA extraction for TREC quantification, and the second one was individually (without pooling) subjected to RNA extraction for *Ptk7* and viral RNA quantification.

### T Cell Receptor Rearrangement Excision Circle Quantification

#### Cell Preparation and Lysis

A pool of a minimum of three thymuses or spleens from mice of the same litter was mechanically digested, and the suspension was pulsed by pipette to remove clumps. The suspension was then washed with cold PBS [with 2% fetal calf serum (FCS)] and filtered through a 70-μm MACS SmartStrainer (Miltenyi Biotec). The obtained cells (thymocytes or splenocytes) were then counted with Malassez cell with trypan blue exclusion of dead cells.

To release their DNA content, 10^5^ cells were lysed in lysis buffer composed of Tris-HCl (10 mM; pH 8.3), Tween 20 (0.05%), Igepal (0.05%), and proteinase K (100 μg/ml) during 30 min at 56°C. Proteinase K was then inactivated at 95°C during 10 min, and samples were stored at −20°C until proceeding.

#### Pre-PCR

sj and DβTRECs were co-amplified together with the CD4 gene used as a single copy gene in a step called “pre-PCR,” using outer (out) primers (Table 1). Briefly, 10 μl of cell lysates or plasmids, containing sjTRECs (or DβTRECs) with CD4 inserts, were added to 90 μl of a mix composed of 1 μl of each primer at 100 mM, 20 μl of 5× colorless GoTaq® Flexi buffer (Promega), 14 μl of MgCl_2_ (25 mM, Promega), 4 μl of deoxynucleoside triphosphate (dNTP, 10 mM, Promega), 0.8 μl of GoTaq® Flexi DNA Polymerase (4U, Promega), and completed with nuclease-free water (Ambion). Amplification was performed in an iCycler thermocycler (Bio-Rad), and the PCR conditions were: initial denaturation at 95°C for 10 min; 22 cycles of amplification each consisting of 30 s at 95°C, 30 s at 60°C, and 2 min at 72°C, followed by final elongation at 72°C for 10 min and cooling at 20°C.

**Table 1:** Primer sequences used in pre-PCR (Out) and T cell receptor rearrangement excision circle quantification by multiplex real-time PCR (In).

**Name**	**Sequences**
CD4 1 Out	CCAACCAACAAGAGCTCAAGGA
In	AGCTCAAGGAGACCACCATGT
CD4 2 Out	CCCAGAATCTTCCTCTGGT
In	TGGTCAGAGAACTTCCAGGT
Jα61 Out	AACTGCCTGGTGTGATAAGAT
In	GGAGTATCTCTTTGGAGTGA
Jα58 Out	CCCAGGACACCTAAAAGGAT
In	AACTCGCACAGTGGAGGAAA
REC1 Out	AGTGTGTCCTCAGCCTTGAT
In	GAAAACCTCCCCTAGGAAGA
Dβ1 Out	TATCCACTGATGGTGGTCTGTT
In	GACGTTGGCAGAAGAGGATT
Jβ1.1 Out	CATGTTTGACATTGCCACAAGT
In	AGCGATTACTCCTCCTATGGT
Jβ1.2 Out	CTCTCTTCACCCCTTAAGATT
In	GTAAAGGAACCAGACTCGTT
Jβ1.3 Out	TGAGGCTGGATCCACAAAGGT
In	TCAAGATGAACCTCGGGTGGA
Jβ1.4 Out	GGGCCATTAGGAAACGTGAT
In	GCAGGAAGCATGAGGAAGTT
Jβ1.5 Out	GGAGGAAGGAAGGATGGTGA
In	CAGAGTCCTGCCTCAAAGAA
Jβ1.6 Out	CCTGTGACATGCCTCATGGTA
In	TCAGGTCTCAGGGATCTAAGA

### sj and DβTREC Quantification

Total sjTREC number was estimated by quantification of δREC1 rearrangement with jα61 and jα58 segments since they represent almost 100% of sjTREC frequency in mice (30). DβTREC content was measured by quantifying Dβ1 rearrangements with Jβ1.1 to 1.6. The CD4 gene was used as a single copy gene, allowing estimation of the number of cells (each cell possesses two alleles of the CD4 gene, so the number of cells is equal to the number of CD4 copies divided by 2). The relative numbers of copies of CD4 gene and TRECs were obtained by multiplex nested qPCR quantification by comparison with plasmids containing both CD4 and sj61 or DJβ4 sequences respectively for sj or DβTREC quantification.

A real-time quantitative PCR assay to detect sj and DβTREC sequences was developed based on a method described by Dulude et al. (30).

Pre-PCR products were diluted 4,000× for CD4, 400× for sj, and 100× for Dβ quantification both for thymus and spleen samples, and relative CD4 and TREC number of copies were determined by qPCR by adding 4 μl of diluted pre-PCR products to 7 μl of Takyon™ No Rox SYBR MasterMix Blue dTTP (Eurogentec), 0.2 μl of CD4 and TREC inner (In) primers (purchased from Eurogentec; Table 1) each at 100 mM, and completed with nuclease-free water to reach a total volume of 14 μl. The amplification program was composed of 5 min at 95°C for initial denaturation; 40 cycles of amplification each consisting of 10 s at 95°C, 15 s at 60°C and 10 s at 72°C, and cooling at 4°C. All reactions were run on a LightCycler 480 thermocycler (Roche Diagnostics) and analyzed using the LightCycler 480 Software by the second derivative max method. Samples were analyzed in triplicate, which never varied by more than 10% from each other, and the average of each sample was used. Standard curves with determined numbers of copies were obtained by performing a 10-fold serial dilution of each plasmid. TREC levels are expressed as TRECs per 10^6^ cells. Copy numbers of TRECs and CD4 were calculated by reporting the coefficient threshold (Ct) of each sample to the standard curve.

### *Ptk7* Gene Expression

#### RNA Extraction

Total RNA was extracted from washed thymuses by the acid guanidium thiocyanate–phenol–chloroform extraction procedure using Tri-Reagent (Sigma, St. Louis, MO, USA), precipitated with isopropanol, and washed with 75% ethanol as described by Chomczynski and Sacchi (31). Sterile nuclease-free water treated under the same extraction procedure served as a negative control. The extracted RNA was dissolved in 30 μl of nuclease-free water (Ambion), quantified using the Nanodrop 2000 (UV-Vis Spectrophotometer; Thermo Scientific, Waltham, MA, USA), and submitted to DNase digestion, at 37°C for 15 min, by DNase type I (Roche) to eliminate contaminating genomic DNA, followed by enzyme heat inactivation at 85°C for 8 min (32). The purified RNA was quantified again and stored at −80°C until use in reverse transcription (RT).

### Complementary DNA Synthesis

RNA was reverse-transcribed using Moloney Murine Leukemia Virus (M-MLV) Reverse Transcriptase (RT, Invitrogen). Complementary DNA (cDNA) synthesis was performed with ~500 ng of RNA in a final volume of 20 μl containing 20 U/μl of RT, 2.5 μM of anchored-oligo(dT)_18_ primer (Roche), 60 μM of random hexamer primer (Roche), 20 U of RNase inhibitor (Promega), and 1 mM each dNTP.

According to the manufacturer's instructions, secondary structures were denatured by heating samples for 10 min at 65°C in the presence of anchored-oligo(dT)_18_ primer, random hexamer primer, dNTP mix, and water PCR grade in a final volume of 20 μl. Samples were then cooled on ice immediately, briefly centrifuged, supplemented with 5X First-Strand Buffer, 0.1 M dithiothreitol (DTT) and 40 U of RNaseOUT™ Recombinant Ribonuclease Inhibitor, and incubated at 37°C for 5 min. One microliter (200 U) of M-MLV RT was then added, and each sample was incubated at 25°C for 10 min followed by 50 min at 37°C. The enzyme was inactivated by heating at 70°C for 15 min. Two negative controls were performed in each reaction, one without RNA and the second without the RT enzyme (RT minus control allowing the detection of eventual genomic DNA contamination).

### Quantitative PCR With SYBR Green

Real-time PCR was performed using 2X Takyon™ No Rox SYBR® MasterMix dTTP Blue (Eurogentec), *Ptk7* forward primer 5′-GCCGTGTGCCTTGAACCTC-3′, and *Ptk7* reverse primer 5′-CTCCACTTCACAACGGAGCA-3′ (Eurogentec). Each reaction consisted of 20 μl containing 1 μl of cDNA, 10 μl of Takyon, and 3 pmol of each primer. The real-time qPCR was run on an iCycler iQ real-time detection system (Bio-Rad) using SYBR green detection. The cycling parameters were 95°C for 10 min, followed by 40 cycles each consisting of denaturation at 95°C for 30 s, annealing at 60°C for 30 s, and elongation at 72°C for 25 s. A melting curve from 55 to 95°C was performed in each PCR reaction. The Ct was defined as the PCR cycle number that crosses an arbitrarily placed signal threshold. All samples were amplified in triplicate, and the mean was used for further analysis. Each PCR reaction also included an RT minus negative control to confirm the absence of genomic DNA and a no-template negative control to check for primer-dimers.

A plasmid standard [128-pcDNA3.1 hygro(+)] containing the target region was generated for each *Ptk7* primer. The amplified products were run on a 1% agarose gel to confirm the specificity of the amplification, and products were cloned using T4 DNA Ligase (Thermo Scientific). Plasmids were isolated using the Wizard® SV Gel and PCR Clean-Up System (PROMEGA, Madison, USA) with DNA concentrations determined by Nanodrop 2000 (Thermo Scientific). Standard curves were obtained by using triplicate of 10-fold dilutions of plasmid DNA. Copy numbers for each reaction were calculated from the standard curves assuming that the average molecular mass of a double-stranded DNA molecule is 650 Daltons.

### Viral Load

One microliter of cDNA (generated from thymuses and spleens as described above) was used to quantify CV-B4 RNA by qPCR with the same program as described for *Ptk7* gene. The primer sequences used were: forward primer EV1 5′-CAAGCACTTCTGTTTCCCCGG-3′ and reverse primer EV2 5′-ATTGTCACCATAAGCAGCCA-3′. Enterovirus 71 RNA control (Vircell, Granada, Spain) was used as a standard for a calibration curve containing five points ranging from 1.26 × 10^4^ to 1.26 copies/ml.

### Statistical Analysis

Statistical analysis was performed with GraphPad Prism 5 software version. The Mann–Whitney test was used for cellularity, size, TREC, and *Ptk7* analysis. A *p* value < 0.05 was considered statistically significant. *p value < 0.05, **p value < 0.01, ***p value < 0.001.

## Results

### Weight and Cellularity of Thymus and Spleen Increased Following *In utero* Coxsackievirus B4 Infection

In a first step, we compared weight and cell content of thymuses and spleens of infected and control animals. Because of the small weight and cellularity, values were also normalized in function of total body weight (relative weight) or organ weight (relative cellularity), respectively.

### Effect of *In utero* Coxsackievirus B4 Infection on Organs' Weight

**Figure 1:**
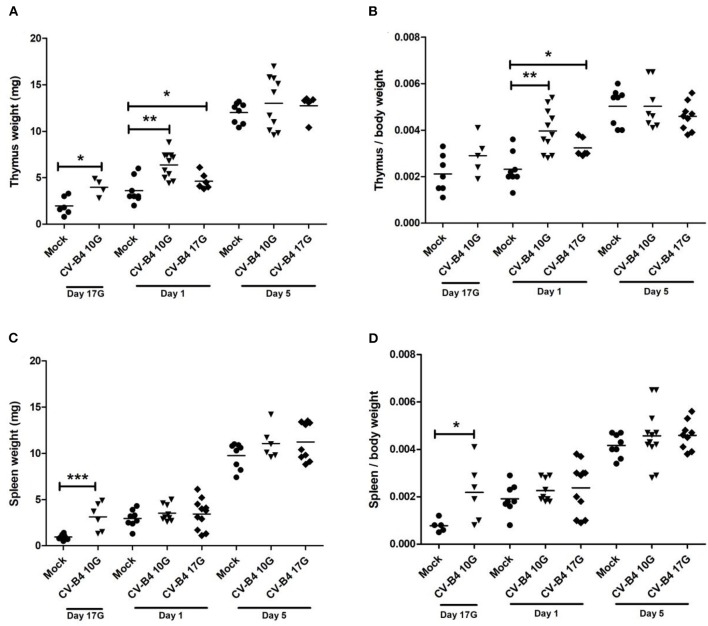
Thymus and spleen weight. Absolute and relative (organ weight/body weight) weights of thymus (**A,B**, respectively) and spleen (**C,D**, respectively) were determined for coxsackievirus (CV) B4-infected and age-matched mock (•) thymuses and spleens, sampled at different time points, from fetuses at day 17 of gestation (day 17G) and newborns at days 1 and 5 from birth. CV-B4 10G (▾): thymus or spleen sampled from mice born to dams inoculated with CV-B4 at day 10 of gestation; CV-B4 17G (♦): thymus or spleen sampled from mice born to dams inoculated with CV-B4 at day 17 of gestation. The Mann–Whitney test was used for statistical analysis. *n* = 4–12 per group ***p < 0.0001, **p < 0.01, *p < 0.05.

In mock-infected mice, thymus absolute weight (in milligrams) increased during development, especially after birth when it tripled (*p* = 0.0005) when comparing days 5–1 (Figure 1A). Following an infection at day 10G, the means of thymus absolute weights were higher in virus-infected compared to mock-infected mice at day 17G (3.975 vs. 1.967 mg, respectively, *p* = 0.019) and day 1 (6.382 vs. 3.6 mg, respectively; *p* = 0.0019). Following inoculation at day 17G, the increase was significant only at day 1 (4.617 vs. 3.6 mg, *p* = 0.04). As shown in Figure 1B, when thymus weight was corrected to matched body weight (relative thymus weight), a significant difference was noted only at day 1, following inoculation at day 10G (4 × 10^−3^ vs. 2.3 × 10^−3^, *p* = 0.0014) as well as day 17G (3.2 × 10^−3^ vs. 2.3 × 10^−3^, *p* = 0.022).

The same profile was observed for the spleen absolute weight (in milligrams) when comparing mock-infected mice at different time points. Indeed, spleen absolute weight increased rapidly during development. At day 5, the weight was 10- and 3-fold higher than at days 17G and 1, respectively (Figure 1C). For mice born to dams inoculated at day 10G, the increase in weight was significant only at day 17G (3.117 vs. 0.95 mg, *p* = 0.0007). On the contrary, no significant difference was observed in spleen weight of mice born to dams inoculated at day 17G (Figure 1C). The relative spleen weight was significantly increased only at day 17G following an inoculation at day 10G when it was ~3-fold higher compared to matched controls (2.2 × 10^−4^ vs. 8 × 10^−4^, respectively, *p* = 0.017). Inoculation at day 17G did not induce any significant change in this parameter, neither at day 1 nor at day 5 (Figure 1D).

### Effect of *In utero* Coxsackievirus B4 Infection on Organs' Cellularity

As described in the *Materials and Methods* section, the number of cells in suspensions obtained from thymus and spleen was counted, and viability was checked by trypan blue staining. Viability was always higher than 95%. Evidently, cellularity followed the same increase in weight, over time, in both tissues.

**Figure 2:**
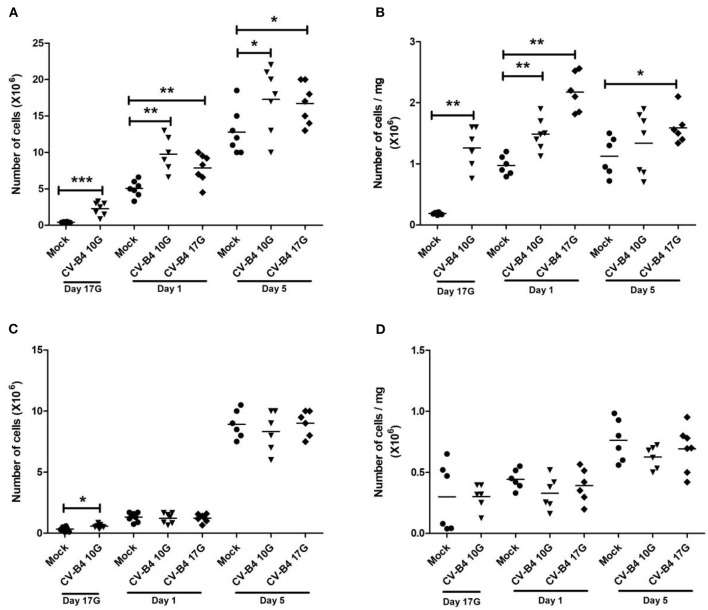
Thymus and spleen cellularity. Absolute and relative (number of cells/mg of cells) numbers of cells in the thymus (**A,B**, respectively) and spleen (**C,D**, respectively) mice assessed by trypan blue exclusion test. Values were determined in coxsackievirus (CV)-B4-infected and age-matched mock (•) thymuses and spleens, sampled at different time points, from fetuses at day 17 of gestation (day 17G) and newborns at days 1 and 5 from birth. CV-B4 10G (▾): thymus or spleen sampled from mice born to dams inoculated with CV-B4 at day 10 of gestation; CV-B4 17G (♦): thymus or spleen sampled from mice born to dams inoculated with CV-B4 at day 17 of gestation. The Mann–Whitney test was used for statistical analysis. *n* = 6–8 per group ***p < 0.0001, **p < 0.01, *p < 0.05.

During development, the number of cells (×10^6^) in the thymus of mock-infected controls increased by 15-fold at day 1 and by 40-fold at day 5 compared to fetuses at day 17G. As previously observed for absolute thymus weight, inoculation at day 10G resulted in a significant increase in absolute cellularity at day 17G (2.28 × 10^6^ vs. 0.42 × 10^6^, *p* = 0.0006), day 1 (9.47 × 10^6^ vs. 5.043 × 10^6^, *p* = 0.0021) and day 5 (17.3 × 10^6^ vs. 12.8 × 10^6^, *p* = 0.041) compared to mock-infected mice. A significant increase in thymus absolute cellularity was equally noted following inoculation at day 17G, both at day 1 (7.87 × 10^6^ vs. 5.04 × 10^6^, *p* = 0.009) and day 5 (16.71 × 10^6^ vs. 12.8 × 10^6^, *p* = 0.02) (Figure 2A). An increase in relative thymus cellularity (cell number divided by thymus weight) was also noted but with a significant difference only at day 17G (1.26 × 10^6^/mg of tissue vs. 0.185 × 10^6^/mg of tissue, *p* = 0.0011) and day 1 (1.48 × 10^6^/mg of tissue vs. 0.97 × 10^6^/mg of tissue, *p* = 0.0012) following an inoculation at day 10G and at both day 1 (2.174 × 10^6^/mg of tissue vs. 0.97 × 10^6^/mg of tissue, *p* = 0.0011) and day 5 (1.6 × 10^6^/mg of tissue vs. 1.125 × 10^6^/mg of tissue, *p* = 0.015) following an inoculation at day 17G (Figure 2B).

The number of cells (×10^6^) in the developing spleen of mock-infected controls was 4- and 30-fold higher at days 1 and 5, respectively, than at day 17G. Following an infection, no significant change was observed in absolute spleen cellularity, except at day 17G in fetuses born to dams inoculated at day 10G (0.58 × 10^6^ vs. 0.335 × 10^6^, *p* = 0.031) (Figure 2C). Relative spleen cellularity seemed slightly lower in CV-B4- than in mock-infected mice, both at days 1 and 5, whatever mice were inoculated at day 10G or at day 17G, and the difference was not significant (Figure 2D).

### sj and DβTRECs Decreased in Both Thymus and Spleen Following *In utero* Coxsackievirus B4 Infection

Intracellular sj and Dβ TREC levels were quantified in thymocytes and in periphery in splenocytes by multiplex qPCR.

**Figure 3:**
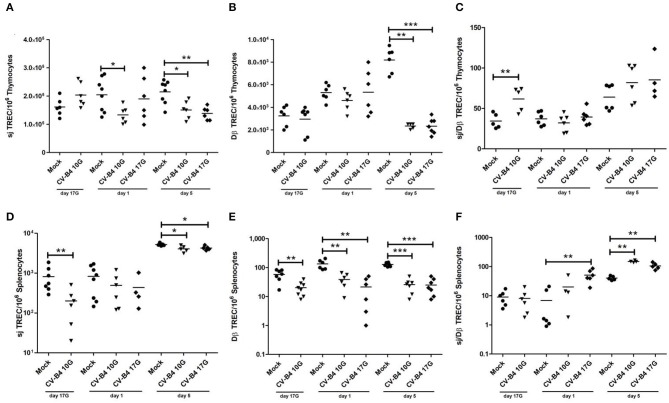
Quantification of T cell receptor rearrangement excision circles (TRECs) as markers of thymopoiesis. The number/10^6^ cells of sjTRECs and DβTRECs, as well as sj/Dβ ratio, were quantified in thymocytes **(A–C)** and splenocytes **(D–F)** of age-matched controls (•) and coxsackievirus (CV) B4-infected thymuses and spleens, sampled at different time points, from fetuses at day 17 of gestation (day 17G) and newborns at days 1 and 5 from birth. CV-B4 10G (▾): thymus or spleen sampled from mice born to dams inoculated with CV-B4 at day 10 of gestation; CV-B4 17G (♦): thymus or spleen sampled from mice born to dams inoculated with CV-B4 at day 17 of gestation. The Mann–Whitney test was used for statistical analysis. *n* = 4–10 per group. ***p < 0.001, **p < 0.01, *p < 0.05. Data for spleen were log-transformed before analysis.

As shown in Figure 3A, the number of sjTRECs/10^6^ thymocytes increased moderately during development in mock-infected mice. An inverted pattern (decrease over time) was observed following CV-B4 infection either at day 10G or 17G. sjTREC frequencies were significantly decreased both at days 1 and 5 (133,700 vs. 204,300, *p* = 0.0147 and 150,900 vs. 214,600, *p* = 0.0147, respectively), following inoculation at day 10G. Following virus inoculation at day 17G, the difference was significant (138,500 vs. 214,600, *p* = 0.0023) only at day 5 after birth, with a decrease by 1.5-fold compared to controls.

As far as DβTREC frequencies in mock-infected control mice, they increased gradually during development (2.5-fold at day 5 compared to day 17G). Analysis of the number of DβTRECs/10^6^ thymocytes in infected animals revealed a significant decrease by 3.5-fold only at day 5 following CV-B4 inoculation either at day 10G or 17G (2,347 vs. 8,190, *p* = 0.0022 and 2,337 vs. 8,190, *p* = 0.0006, respectively) (Figure 3B). Lower values in DβTRECs were equally observed at days 17G and 1 following inoculation at day 10G and at day 1 following inoculation at day 17G but did not reach statistical significance.

The sj/DβTRECs ratio, as an indicator of intra-thymic proliferation, presented the same value at days 17G and 1 with an increase by 2-fold at day 5 (*p* = 0.0022) in mock-infected mice. Following CV-B4 infection, no significant change was obtained compared to control mice, whatever was the inoculation time, except an increase by 2-fold (61.6 vs. 34.32, *p* = 0.0079) at day 17G following inoculation at day 10G (Figure 3C).

In the periphery, the number of sjTRECs/10^6^ splenocytes increased during development, especially at day 5 when it was 5-fold higher compared to days 17G and 1 (Figure 3D). Virus inoculation either at day 10G or at day 17G did not induce any significant effect at day 1. A significant decrease was however observed at days 17G and 5 following inoculation at day 10G (200.7 vs. 817.6, *p* = 0.0041 and 4,080 vs. 5,223, *p* = 0.0139, respectively) and at day 5 following inoculation at day 17G (4,284 vs. 5,223, *p* = 0.0159).

As far as the number of DβTRECs/10^6^ splenocytes in control mice, it increased with development, by 2.5-fold at day 1 and 1.8-fold at day 5 compared to fetuses at day 17G. In infected animals, a significant decrease was noted at the different studied time points, whatever was the inoculation time (Figure 3E). Indeed, compared to mock-infected mice, DβTREC frequencies were more than 3-, 3.5-, and 5-fold lower at day 17G (20.13 vs. 58.14, *p* = 0.0045), day 1 (38.83 vs. 134.2, *p* = 0.0011), and day 5 (25.86 vs. 127.7, *p* = 0.0006), respectively, in mice born to dams inoculated at day 10G. Following inoculation at day 17G, those frequencies were 6.3- and 5-fold lower at day 1 (21.33 vs. 134.2, *p* = 0.0011) and day 5 (25 vs. 127.7, *p* = 0.0006), respectively.

The sj/DβTREC ratio analysis in spleen of mock-infected mice shows that it followed the same profile as for sj and Dβ TRECs, increasing by 5-fold at day 5 compared to day 17G. A significant increase in the sj/DβTREC ratio was observed at day 5 following an inoculation at day 10G (150 vs. 40.5, *p* = 0.0079) and at days 1 and 5 following an inoculation at day 17G (50.86 vs. 6.906, *p* = 0.0022 and 105.3 vs. 40.5, *p* = 0.004, respectively) (Figure 3F).

### *Ptk7* Gene Expression

**Figure 4:**
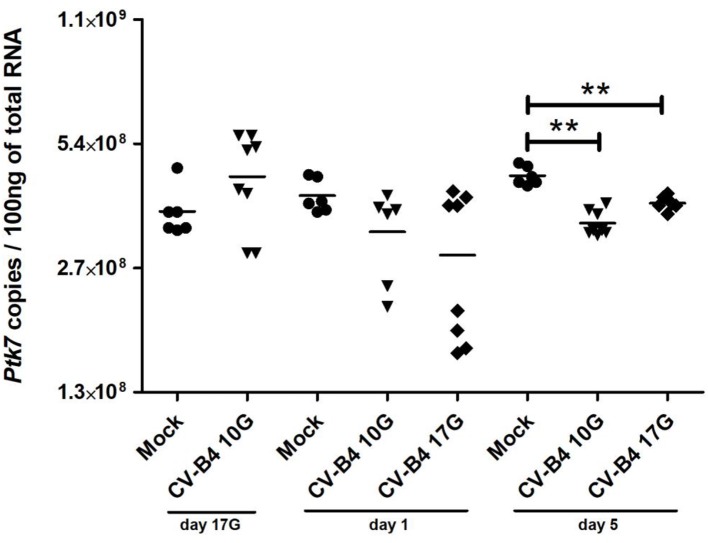
*Ptk7* mRNA expression. RNA was converted by reverse transcription (RT), and the number of copies per 100 ng of RNA was determined in thymocytes of age-matched controls (•) and coxsackievirus (CV) B4-infected mice by using a serial dilution of a reference plasmid. CV-B4 10G (▾): thymus sampled from mice born to dams inoculated with CV-B4 at day 10 of gestation; CV-B4 17G (♦): thymus sampled from mice born to dams inoculated with CV-B4 at day 17 of gestation. The Mann–Whitney test was used for statistical analysis. *n* = 6–8 per group. ***p < 0.001, **p < 0.01, *p < 0.05. Data were log-transformed before analysis.

*Ptk7* expression level per 100 ng of total RNA in control thymuses increased significantly during development at day 1 (*p* = 0.0449) and at day 5 (*p* = 0.015) compared to fetuses at day 17G (Figure 4). It has a tendency to increase at day 17G following CV-B4 inoculation at day 10G, then to decrease at day 1 following inoculation at either day 10G or 17G, even though no statistically significant difference was found. That decrease became significant at day 5 following inoculation at either at day 10G (3.45 × 10^8^ vs. 4.5 × 10^8^, *p* = 0.0012) or day 17G (3.85 × 10^8^ vs. 4.5 × 10^8^, *p* = 0.0025).

### *In utero* Infection of Thymus and Spleen by Coxsackievirus B4

The levels of CV-B4 RNA in offspring's thymuses and spleens were investigated by quantitative RT-PCR.

**Figure 5:**
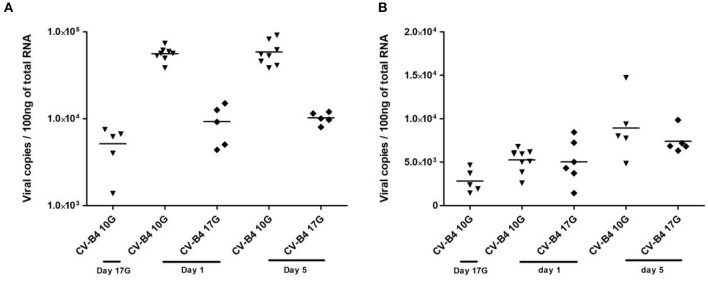
Viral load in infected tissues. Thymocytes and splenocytes were sampled from fetuses at day 17 of gestation (day 17G) and from newborns at days 1 and 5 from birth, following an infection at day 10 or 17 of gestation (day 10G ▾ or 17G♦). Total RNA was extracted, converted to cDNA, and the level of viral RNA in the thymocytes **(A)** or splenocytes **(B)** was determined by qPCR and expressed as copy number per 100 ng of total RNA. *n* = 5–9 per group. Coxsackievirus (CV) B4 10G (▾): thymus sampled from mice born to dams inoculated with CV-B4 at day 10 of gestation; CV-B4 17G (♦): thymus sampled from mice born to dams inoculated with CV-B4 at day 17 of gestation. Data were log-transformed before analysis.

When the inoculation was performed at day 10G, 85% of thymuses were positive, while about 40% of thymuses were positive following inoculation at day 17G. Most elevated viral loads were recorded in samples at days 1 and 5 from offspring born to dams inoculated at day 10G, ranging between 4 × 10^4^ and 9 × 10^4^ copies per 100 ng of total RNA (Figure 5A). Thymuses sampled from fetuses presented a lower level of CV-B4 RNA, ranging from 2,073 to 8,215 copies, comparable to those from offspring born to dams inoculated at day 17G and sampled at day 1 or 5.

Similarly to the thymus, ~78% of spleens sampled from offspring born to dams inoculated at day 10G were positive against only 33% when inoculation was performed at day 17G. The viral load in the spleen was lower and almost the same following CV-B4 inoculation either at day 10G or 17G, ranging from 1,440 to 8,440 copies, with only a slight increase at day 5 when it reached 14,707 copies per 100 ng of total RNA (Figure 5B).

Intriguingly, by using the Spearman's test, we could not evidence any eventual correlation between the viral load in the infected tissues and the observed variations among the different analyzed parameters (weight, cellularity, TRECs, and Ptk7 transcripts).

## Discussion

Studies focusing on viral infection of the fetal thymus are still very rare. We have previously shown that CV-B4 reaches this tissue in the course of an *in utero* infection and that such an infection disturbs T cell differentiation (21). In this study, thymic function was explored in pregnant Swiss albino (an outbred strain which more accurately reflects natural variations inside a population) mice that were orally inoculated by CV-B4 E2 at two different time points. As previously explained (33), the CV-B4 E2 strain that we used was isolated by Yoon et al. (34) but shares 99% homology with the E2 plaque-purified variant of the CV-B4 Edwards strain already isolated by Kibrick and Benirschke (35) from the myocardial tissue of a neonate who suffered from encephalohepatomyocarditis (with focal necrosis and inflammation of the pancreas) and died 36 h after birth. The virus was vertically transmitted from mother to the infant based on serological and virological evidence.

As described in the literature, the time of inoculation during pregnancy influences the outcome of viral infection [as discussed in our previous paper (36)]. We choose to inoculate mice during the second (at day 10G) and the third week (at day 17G) of gestation, as we previously showed that inoculation at those two different times induces significant changes in T cell populations (21).

A wide array of immunological, cell surface, and molecular markers was used for the assessment of thymic function (37). Before delving into the molecular level, thymic function was simply assessed through thymus and spleen weight and cellularity. The choice of the spleen relies on the fact that it is the most important secondary lymphoid organ and since it is the simplest and easiest to sample at this early stage of development. Thus, we observed that following *in utero* infection by CV-B4, the thymus and spleen have grown in size and an increase in cellularity was noted regardless the time of inoculation. This suggests that CV-B4 infection induces thymic and peripheral cellular proliferation and accumulation.

TRECs are generated as a by-product of the TCR rearrangement process in the thymus and are enriched in newly generated T cells (4). sj and DβTREC quantification was the preferred tool as a surrogate marker for thymic export (7). DβTRECs are precisely created at the DN2 stage, just before the intensive T cell proliferation phase, and then, between the DN4 stage and the DP stage, occurs the production of sjTRECs. Accordingly, sj/DβTRECs ratio calculation allows the assessment of intrathymic proliferation (30). Furthermore, in the study conducted by Dulude et al. (30), a strong positive correlation was shown between the level of proliferation of the DN4 cell subset in the thymus and the peripheral sj/DβTREC ratio.

Non-monotonic dynamics of the data is hard to interpret. sj and DβTREC analysis in offspring showed a significant decrease following CV-B4 infection compared to controls, a decrease that gets worse as time goes by, both in the thymus and in the spleen (periphery).

That decrease in TRECs at the thymus level vs. an increase in cellularity may obviously be explained by an increased proliferation and accumulation of thymocytes. Intriguingly, the ratio of sj/Dβ being comparable to that in controls, we should take more caution and consider several nonexclusive explanations: (i) increased proliferation with a disturbance in TCR chain rearrangement [temporary shift or rearrangement did not achieve (did not occur at all, or was not successful in generating TRECs, due to any blockade)] (ii) increased proliferation with a disturbance in the maturation of thymocytes [mainly a decrease in the DN subpopulation, as observed at days 1 and 5 from birth, in our previous study (21)], (iii) a blockade in thymic export, and (iv) an increase in the recruitment of early thymic progenitor (ETPs) from BM and liver to the thymus. During fetal life, in addition to the BM, the liver provides T cell precursors that colonize the fetal thymus where their maturation/differentiation takes place (38).

In the periphery, the detection of sjTRECs (markers of naive T cells leaving the thymus, also called RTE) indicates thymic export. Contrary to the thymus, a decrease of sj and Dβ TRECs in the spleen vs. an expansion of the size can be explained by an activation of cell proliferation following an infection. The sj/Dβ TREC ratio confirms this hypothesis since it increased following an infection compared to controls, with a positive correlation with the viral load. This is in line with a previous suggestion that a defect in thymic T cell production may be compensated by increased self-replication of peripheral T cells (11). This phenomenon consists of immune recovery through peripheral T cell proliferation, therefore, recovery of thymic function, and it was referred to as thymic rebound (39).

TREC assessment in the periphery was firstly conducted in peripheral blood pooled from all mice of the same litter. Additionally to the fact that at such an early age of life, blood collection is not easily feasible, results of some analyzed samples at days 1 and 5 showed a big discrepancy between samples in the same conditions (results not depicted). A study conducted by Elgbratt et al. (40), in colonic mucosa of patients with ulcerative colitis, revealed that assessment of TREC levels in peripheral blood is not informative.

The thymus is considered as a target organ for some viral infections. Those infections can induce heavy consequences such as a significant change in thymic weight and structure and interference with thymic activity [reviewed by Nunes-Alves et al., (41)]. A reduction in TREC level following human immunodeficiency virus (HIV) (42), hepatitis C virus (HCV) (43), and cytomegalovirus (CMV) (44) infections was previously described. Increasing thymic volume together with a low sjTREC level were described in peripheral blood mononuclear cells (PBMCs) of HIV-positive patients after interruption of the highly active antiretroviral therapy (45). Patients with higher thymic volume had higher plasma viral load and a greater decline in CD4^+^ cell count compared to patients with a lower viral load. This was suggested as one of HIV strategies to increase the number of available target cells (45). A significant reduction of RTE, as assessed by coding joint (cj)TREC quantification, was described in 14 patients with HCV infection compared to age-matched controls (43). That deficit in RTE limits generation and maintenance of a diverse TCR repertoire that is unable to provide sufficient diversity for monitoring the infection and suppressing of the virus (46). Low sjTREC values in PBMCs and higher CMV loads were reported in patients with CMV infection (44). Presence of autoreactive T cells in the periphery was associated with both CMV infection and autoimmune diseases, namely, RA, in the same patients (15).

Since *Ptk7* expression declines with maturation and is no longer expressed in RTEs, so it is judicious to analyze *Ptk7* transcripts just in the thymus. The increase in *Ptk7* gene expression matches up with thymus size and development in mock-infected mice, which is considered as an indicator of a balance between T cell production and export. Absolute quantification revealed a decrease in the expression of *Ptk7* gene following an *in utero* CV-B4 infection. This reinforces the hypothesis of a deficiency in the export of cells in a mature stage (SP4 and SP8). This is in accordance with conclusions recently drawn from our previous study (21) showing an increase in SP4 at day 1 and in both SP4 and SP8 at day 5 following CV-B4 infection.

The opposite effect recorded at day 17G in the thymus for sj and *Ptk7*, after inoculation at day 10G, is evocative of what we previously observed with thymocyte subpopulations (21). In that previous study, *in utero* infection by CV-B4 induced blockade of DN to DP passage at day 17G. This can be explained by the fact that sjTREC molecules are more precisely produced at DN4 to DP transition (30), and it would be interesting to characterize DN T cells. This is in line with our current study since *in utero* infection of the thymus resulted in a nonsignificant increase of *Ptk7* gene expression in fetal thymus, a gene mainly overexpressed by DN T cells. Let us remember that *Ptk7* gene expression declines progressively with maturation until its repression in SP T cells in the thymus and shortly after exportation in RTE SP4 T cells (8–10).

Phenotypic characterization of T lymphocytes in the periphery, precisely in the spleen, was assessed by flow cytometry by using previously established phenotypic characteristics of naive and memory T cells, identified as CD62L^+^CD44^−^ or CD62L^−^CD44^+^, respectively, for both SP4 and SP8. However, as shown by TREC quantification, the number of T cells in the periphery was very low, which prevented us from phenotyping and quantifying both different cell categories (data not shown).

*In utero* infection of the spleen by CV-B4 was explored for the first time. The less important effect recorded within the spleen may be explained by lower viral loads.

Autoimmune disorders have a multifactorial etiology with genetic, immunological, hormonal, and environmental factors (47). A decrease in the influx of RTEs, therefore, a lower level of TREC molecules, has been proposed as one of putative factors that can lead to the pathogenesis of autoimmune diseases (48). Indeed, a reduced thymic output vs. an excessive proliferation of peripheral T cells was described in patients with acquired RA (11, 12). Similar immunologic features were noted in SLE patients by Kayser et al. (16). Indeed, patients with active SLE presented a lower TREC levels in peripheral T cells, but not those with quiescent disease (17). In patients with primary progressive multiple sclerosis, reduced TREC level and thymic output were observed and resulted in peripheral immune alterations and clonal T cell expansions (49).

Abnormal T cell population dynamics can be a common mechanism in autoimmune diseases and may be attributed to viral infections (50). CV-B4 are very likely implicated in T1D (51) and dilated cardiomyopathy (52). Both diseases are T cell mediated and characterized by the recruitment of cytotoxic T cells directed to CV-B antigens on the surface of β cells and myocytes, leading to inflammation and organ injury (53–55). Following infection, an increase in thymus weight and cellularity vs. a low TREC level, could interfere with thymocyte maturation and lead to escape from apoptosis resulting in a premature release of T cells in the periphery. This may increase the possibility of impaired selection and circulating of autoreactive T cell clones which may hinder the maintenance of immune tolerance (48).

In conclusion, our results (summarized in Table 2) show a significant decrease in TREC level in the thymus and spleen (at days 1 and 5 from birth) following an *in utero* infection by CV-B4. This infection also resulted in an increase in thymus size, which could be explained by thymocyte proliferation but with a disturbance in their TCR rearrangement and in their maturation. Accumulation of thymocytes (mainly immature DP cells, as suggested by the level of *Ptk7* transcripts), reflecting a defect in thymic export, should equally be considered. The increase in spleen size, for its part, seems rather to be due to an excessive proliferation as demonstrated by sj/Dβ TREC ratio. Reduced thymic output and enhanced peripheral T cell turnover could be one of the putative factors implicated in CV-B-mediated automimmune pathogenesis. Disturbance in T cell homeostasis may contribute to disequilibrium between polyclonal and naive T cells and possibly to the presence of oligoclonal memory autoreactive T cells which may interfere with the maintenance of immune tolerance and lead to autoimmune diseases.

**Table 2:** Summary of significant differences (vs. mock) recorded for each of the different studied parameters.

**Sampling**		**Thymus**	**Spleen**	**Interpretation**
Day 17G		↑ Weight^*^ ↑ Cellularity^***^ and R. cellularity^**^ ↑ sj/DβTREC^**^	↑ Weight^***^ and R. weight^*^ ↑ Cellularity^*^ ↓ sj TREC^**^ ↓ Dβ TREC^**^	Increased proliferation and accumulation of thymocytes vs. decreased RTE and increased proliferation in the periphery
Day 1	Inoculated at day 10G	↑ Weight^**^ and R. weight^**^ ↑ Cellularity^**^ and R. cellularity^**^ ↓ sj TREC^*^	↓ Dβ TREC^**^	Disturbance in the maturation of thymocytes and blockade in thymic export vs. increased proliferation in the periphery
	Inoculated at day 17G	↑ Weight^*^ and R. weight^*^ ↑ Cellularity^**^ and R. cellularity^**^	↓ Dβ TREC^**^ ↑ sj/Dβ TREC^**^	Accumulation of thymocytes vs. increased proliferation in the periphery
Day 5	Inoculated at day 10G	↑ Cellularity^*^ ↓ sj TREC^*^ ↓ Dβ TREC^**^ ↓ Ptk7^**^	↓ sj TREC^*^ ↓ Dβ TREC^***^ ↑ sj/Dβ TREC^**^	Disturbance in the maturation of thymocytes and blockade in thymic export vs. decreased RTE but increased proliferation in the periphery
	Inoculated at day 17G	↑ Cellularity^*^ and R. cellularity^*^ ↓ sj TREC^**^ ↓ Dβ TREC^***^ ↓Ptk7^**^	↓ sj TREC^*^ ↓ Dβ TREC^***^ ↑ sj/Dβ TREC^**^	Disturbance in the maturation of thymocytes and blockade in thymic export vs. decreased RTE but increased proliferation in the periphery

*** *p < 0.001*,

** *p < 0.01*,

* *p < 0.05. RTE, recent thymic emigrant; TREC, T cell receptor rearrangement excision circle*.

## Data Availability Statement

All datasets generated for this study are included in the article/supplementary material.

## Ethics Statement

The animal study was reviewed and approved by Faculty of pharmacy of Monastir, University of Monastir.

## Author Contributions

AH conceptualized the study and prepared the manuscript. HJm helped in mice husbandry, inoculation, and dissection. AH, HMi, GB, and CR performed the experiment. HMa, GB, and DH helped, analyzed, and interpreted the data. HJa, MA, and VG supervised the study and corrected the manuscript. All authors read and approved the final manuscript.

### Conflict of Interest

The authors declare that the research was conducted in the absence of any commercial or financial relationships that could be construed as a potential conflict of interest.
